# The N1038S Substitution and ^1153^EQTRPKKSV^1162^ Deletion of the S2 Subunit of QX-Type Avian Infectious Bronchitis Virus Can Synergistically Enhance Viral Proliferation

**DOI:** 10.3389/fmicb.2022.829218

**Published:** 2022-03-30

**Authors:** Shuyun Li, Shunyi Fan, Nianning Li, Yuxi Shen, Xuelian Xiang, Wen Chen, Jing Xia, Xinfeng Han, Min Cui, Yong Huang

**Affiliations:** College of Veterinary Medicine, Sichuan Agricultural University, Chengdu, China

**Keywords:** infectious bronchitis virus, infectious clone, S2 subunit, replication, pathogenicity

## Abstract

The S2 subunit of infectious bronchitis virus (IBV) plays a critical role in the process of IBV infection. A comparison between the S2 subunit sequence of chicken embryo kidney cell (CEK) adapted virulent QX-like IBV strain SczyC30 (hereafter referred to as zy30) and its CEK-attenuated strain, SczyC100, revealed an N1038S substitution in S2 subunit and a ^1154^EQTRPKKSV^1162^ residue deletion in the C-terminus of the S2 subunit. In order to explore whether these two mutations are related to changes in the biological characteristics of IBV, we firstly constructed an infectious clone of zy30 using a bacterial artificial chromosome (BAC), which combines the transcription of infectious IBV genomic RNA in non-susceptible BHK-21 cells with the amplification of rescued virus rzy30 in CEK cells. Then, three recombinant viruses, including an rzy30S2-N1038S strain that contained the N1038S substitution, an rzy30S2-CT9^△^ strain that contained the ^1154^EQTRPKKSV^1162^ deletion, and an rzy30S2-N1038S-CT9^△^ strain that contained both mutations, were constructed using rescued virus rzy30 as the backbone. The results showed that each mutation did not significantly affect the replication titer in CEK cells but reduced pathogenicity in chickens, while in combination, the N1038S substitution and ^1154^EQTRPKKSV^1162^ deletion improved the proliferation efficiency in CEK cells and reduced pathogenicity, compared to rzy30 strain. The contribution made by the ^1154^EQTRPKKSV^1162^ deletion in reducing pathogenicity was higher than that of N1038S substitution. Our results revealed that the N1038S substitution and ^1154^EQTRPKKSV^1162^ deletion in S2 subunit were deeply involved in the replication efficiency of IBV and contributed to reduction of viral pathogenicity.

## Introduction

Infectious bronchitis virus (IBV) is a group of isolates within the species Avian coronavirus, subgenus *Igacovirus*, genus *Gammacoronavirus*, subfamily *Orthocoronavirinae*, within the family *Coronaviridae*.^1^ It is an etiological agent of infectious bronchitis (IB), which causes severe economic losses in the poultry industry worldwide (Franzo et al., 2019). IBV has a positive-stranded, non-segmented RNA genome of approximately 27.6 kb in length. Generally, IBV isolates are diverse in pathogenicity and antigenicity (Franzo et al., 2017; Li et al., 2019). The emergence of new serotypes and genotypes distinct from traditional vaccine strains enhances the worldwide distribution of IBV and complicates the control of IB (De Wit et al., 2011; Zhang et al., 2015). In China, QX-like IBV remains the dominant circulating genotype, and immune failure frequently occurs due to key antigenic differences between the vaccine strain and field isolates (Fan et al., 2019; Zhao et al., 2019a). Hence, it is of great importance to explore the pathogenic mechanisms of IBV and generate appropriate attenuated vaccines by reverse genetics technology accordingly.

Reverse genetics technology offers a powerful tool to facilitate a greater understanding of the molecular biology and pathogenesis of IBV. To date, the most commonly used methods to obtain the full-length infectious cDNA clones of IBV are based on *in vitro* ligation of cDNA fragments and the use of vaccinia virus as a vector for the propagation of IBV full-length cDNAs (Casais et al., 2001; Zhou et al., 2013; Zhao et al., 2019b). However, both approaches are faced with technical difficulties, including the selection of suitable restriction enzymes, the integrity of IBV full-length genomic RNA produced through *in vitro* transcription, and poor transfection efficiency of large-sized genomic RNA. Moreover, IBV genome toxicity and instability in *Escherichia coli* make it challenging to construct a reverse genetics system for IBV. Bacterial artificial chromosomes (BACs), which are maintained as a single copy in *E. coli*, have been utilized in reverse genetics systems for large RNA viruses, including coronavirus (Almazan et al., 2013; Ye et al., 2020). This approach overcomes the difficulties associated with the instability in *E. coli* of plasmids containing full-length viral cDNA.

IBV contains envelope-anchored spike proteins that mediate viral entry into host cells (Zou et al., 2010; De Wit et al., 2011). The S protein is secreted from the endoplasmic reticulum (ER), firstly as a monomer, which then becomes a homo-trimer in order to enter the Golgi complex (Bosch et al., 2003). In the ER-Golgi Compartment (ERGIC), it is modified by N-linked mannose-rich oligosaccharides. The S protein has two subunits, S1 and S2, which are metastable prior to fusion and are noncovalently linked (Parsons et al., 2019). The S2 protein participates in membrane fusion and its cytoplasmic tail is conserved across genera. Although the S1 subunit, which contains the receptor binding domain (RBD), is necessary for binding to the host receptor (Bouwman et al., 2020), the S2 subunit appears to be the determinant that enhances viral infectivity to susceptible cells *in vitro*. For example, the ability of the IBV Beaudette strain to grow and produce infectious progeny virus in Vero cells is conferred by just 3 amino acids (aa) surrounding the S2’ cleavage site (R687, K689, and L692; Bickerton et al., 2018). The L581F and V617I amino acid substitutions of the S2 subunit play an indispensable role in the adaptation of IBV to chicken kidney (CK) cells (Jiang et al., 2020). In addition, it has been demonstrated that the virulence genes of IBV include S, 3, 5, and replicase genes (Zhao et al., 2019a,b). Therefore, the S2 subunit, which affects the replication characteristics of IBV in the cell, may also have influence on the virulence, which has not yet been confirmed.

An attenuated IBV strain can be obtained by serial passage of a virulent strain in chicken embryo kidney (CEK) cells, although its infectivity to cells is usually enhanced. However, the underlying mechanism of this phenotypic change is unclear. In our laboratory, an attenuated SczyC100 strain with increased cellular infectivity and no pathogenicity to chickens has been obtained by 100 times passage of QX-Type IBV strain Sczy3 in CEK cells (Xia et al., 2018). Sequence analysis showed that there are two site differences in the S2 subunit between SczyC30 and the CEK-attenuated strain SczyC100, whereas the role of these two sites has not been elucidated. In this study, we described a modified BAC-based reverse genetics system for the QX-like IBV SczyC30 strain. To investigate the role of the site differences in replication and attenuation, recombinant strains containing the mutations in S2 subunit were constructed using SczyC30 strain cDNA clone as the backbone. For each recombinant strain, the growth curve in CEK cells was calculated and the pathogenicity in Specific-Pathogen-Free (SPF) chickens was determined.

## Materials and Methods

### Virus, Cell, and Eggs

The China/Sichuan/QX-like/Sczy3/200904 strain (Sczy3, GenBank JF732903) was isolated from IBV infected broiler chickens (Sichuan province, China) in 2009 and found to be a GI-19/QX-like IBV (Zou et al., 2010; Xia et al., 2016). The SczyC30 strain (hereafter referred to as zy30) was obtained by passaging 30 times of Sczy3 strain in CEK cells and sequenced. An attenuated SczyC100 (hereafter referred to as zy100) strain was obtained by 100 times passage of Sczy3 strain in CEK cells. CEK cells were prepared and cultured as previously described (Xia et al., 2016). CEK and BHK-21 cells were maintained in DMEM containing 10% fetal bovine serum (FBS; Tianhang Co., Ltd., Hangzhou, China), penicillin (100 U/ml), and streptomycin (100 mg/ml). SPF chicken embryos were purchased from the Beijing Merial Vital Laboratory Animal Technology Co., Ltd. (Beijing, China) and hatched in our laboratory.

### Plasmids and Bacterial Strains

The pBeloBAC11 plasmid (New England Biolabs Inc., Beijing, China) was used to assemble the IBV infectious cDNA clone. It is a low-copy-number BAC plasmid (i.e., 1 to 2 copies per cell) that allows the stable maintenance of large DNA fragments in DH10B Competent *E. coli* (New England Biolabs Inc., Beijing, China). In this study, all BAC plasmids were extracted and purified with the BAC/PAC DNA Maxi Kit (OMEGA bio-tek, Guangzhou, China) according to the manufacturer’s instructions. The plasmid pcDNA3.1(+) (Thermo Fisher Scientific Inc., Shanghai, China) was used to express the N gene.

### Assembly of the Genomic cDNA of IBV zy30 Strain in the BAC

Viral RNA was extracted using TRIzol reagent (Takara Bio-Inc., Dalian, China) and the cDNA was obtained using a reverse-transcriptase (RT) kit (Takara Bio-Inc., Dalian, China) according to manufacturer’s instructions. After analyzing the full-length genome sequence of the IBV zy30 strain, 7 overlapping fragments (IBV-1, IBV-2, IBV-3, IBV-4, IBV-5, IBV-6, and IBV-7) covering the full genomic cDNA sequence of the zy30 strain were amplified using seven pairs of primers (F1 and R1, F2 and R2, F3 and R3, F4 and R4, F5 and R5, F6 and R6, and F7 and R7). The CMV (cytomegalovirus) promoter fragment was obtained by amplifying the pcDNA3.1(+) plasmid using primers CMVF and CMVR. The hepatitis delta virus (HDV) ribozyme and the bovine growth hormone (BGH) termination sequence were artificially synthesized (Sanggong Biotechnology, Shanghai, China) and named as HB, then amplified using the HBF and HBR primers. Each PCR fragment contained a matched homologous arm sequence (19–22 bp; Table 1).

**Table 1:** Primers used in this study.

Fragments	Length (position, nt)	Primers	Sequences (5′–3′)^a^
IBV-1	520 bp (1–520)	F1	*AATTCGAGCTCGGTACCCGGG*CGTACGACTGAAAATAGATATTATTATATATC
		R1	*CCTGCAGGTCGACTCTAGA*GGATCCCACTGTCTATTGTATGTCTGCTC
IBV-2	4,835 bp (498–5,332)	F2	GAGCAGACATACAATAGACAGTG
		R2	*TGCAGGTCGACTCTAGA*GGATCCAGTACCGTCCACCCATATTGCCTT
IBV-3	3,680 bp (5,311–8,990)	F3	GCAATATGGGTGGACGGTACTGC
		R3	*ATGCCTGCAGGTCGACTCTAGA*GGATCCCTATCACATGACGTGGACAGTA
IBV-4	4,283 bp (8,970–13,252)	F4	CTGTCCACGTCATGTGATAGGTAAG
		R4	*ATGCCTGCAGGTCGACTCTAGA*GGATCCACCTATCGTCGACACAATCTC
IBV-5	5,726 bp (13,231–18,956)	F5	GAGATTGTGTCGACGATAGGTG
		R5	*ATGCCTGCAGGTCGACTCTAGA*GGATCCTGAACAAGCAGATGCGATG
IBV-6	4,355 bp (18937–23,291)	F6	CATCGCATCTGCTTGTTCAGAATG
		R6	*CTGCAGGTCGACTCTAGA*GGATCCCTCTACCATTAACAGGCACTATT
IBV-7	4,323 bp (23,272–27,657)	F7	GTGCCTGTTAATGGTAGAGGTAT
		R7	*ATGCCTGCAGGTCGACTCTAGA*GGATCCTCTAACTCTAAACTAGCCTATA
IBV-8	2,959 bp (17,390–20,348)	F8	*TGCTAAGCCTTGGCATGTTATAAGAC*
		F9	*GCACTCACTGAAACAACTTAATGCAAGTTTAAA**CGTAC*GCCGTTCCTTAGTAGATTAA
HB^b^	341 bp	HBF	*CTAGTTTAGAGTTAGAGCAAAAAAAAAAAAAAAAAAAAAAAAAAAAAA*TGGCCGGCATGGTCCCAG
		HBR	*ATGCCTGCAGGTCGACTCTAGA*GGATCCCATAGAGCCCACCGCATCCCCA
CMV^c^	508 bp	CMVF	*AATTCGAGCTCGGTACCCG*GGATCCGTTACATAACTTACGGTA
		CMVR	GTAGCTCTGCTTATATAGACC
N	1,765 bp	NF	*GCTGGCTAGCGTTTAAACTT*ATGGCGAGCGGTAAGACAACTG
		NR	*TGGATCCGAGCTCGGTACCA*TTTTTTTTTTTTTTTTTTTTTTTTTTTTTTGCTCTAACT

a *The italics represent homologous arm sequences and the underscores represent restriction sites (*BamHI*: GGATCC; *PfI23II*: CGTACG)*.

b *HB: the fragment contained hepatitis delta virus (HDV) ribozyme and the bovine growth hormone (BGH) termination sequence*.

c *CMV: CMV promoter*.

**Figure 1:**
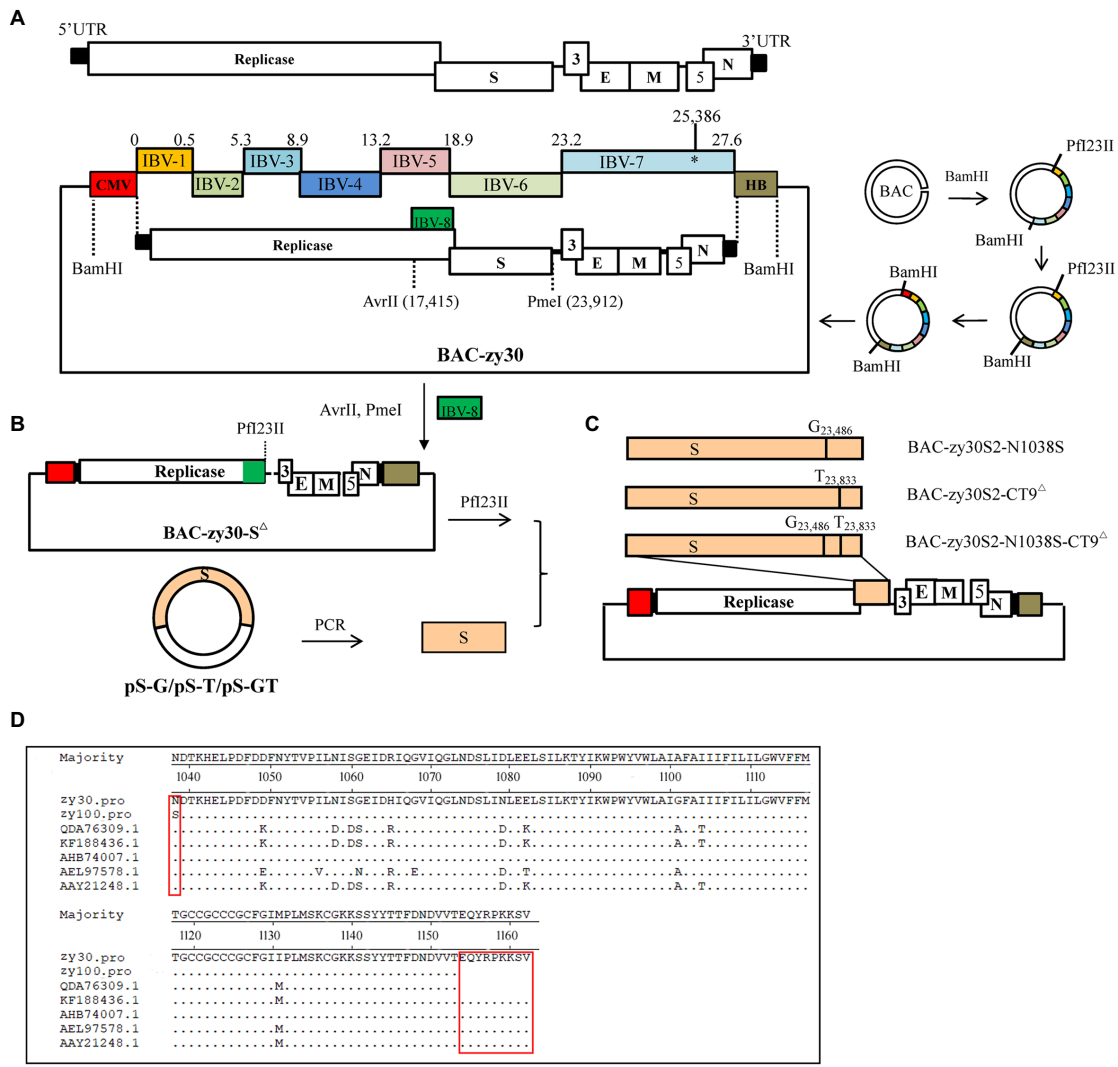
Schematic diagram of the assembly of the full-length IBV cDNA in a BAC plasmid. **(A)** Genome schematics showed the length of 27 kb for the IBV zy30 strain. The regions coding for the replicase polyproteins, the structural proteins spike (S), envelop (E), matrix (M), and nucleocapsid (N) proteins, and the accessory proteins (3a, 3b, 5a, and 5b) were also indicated. UTR: untranslated regions. The IBV genomic cDNA sequence of IBV QX-like zy30 strain was assembled in plasmid pBeloBAC11 by sequentially cloning PCR fragments IBV-1 to IBV-7 using the *BamHI* restriction enzyme. Then, the HB fragment containing hepatitis delta virus (HDV) ribozyme and the bovine growth hormone (BGH) termination sequence was cloned behind zy30 3ʹ terminal using the *BamHI* restriction enzyme, and the CMV promoter was cloned to the front of the zy30 5ʹ terminus using the *PfI23II* restriction enzyme. **(B)** The intermediate plasmid BAC-zy30-S^△^ with complete S gene sequence deletion and plasmids PS-G, PS-T, and PS-GT with different substitutions in the S gene were constructed. **(C)** The three mutated S gene fragments were introduced into the BAC-zy30-S^△^ plasmid, to assemble three full-length genomic cDNA sequences containing the different mutant S genes. **(D)** Alignment of the S2 subunit between IBV strains Beaudette (GenBank accession number AAY21248), M41 (QDA76309.1), 4/91 (AEL97578.1), IBYZ (AHB74007.1), H120 (KF188436), zy30, and zy100 were shown. The amino acid differences were labeled with a red box.

Two restriction enzymes, *BamHI* and *PfI23II*, were used for the construction of the recombinant BAC plasmid containing the full cDNA sequence of the zy30 strain (Figure 1A). The *BamHI* restriction site occurs singularly in the BAC plasmid but is absent from zy30 genomic cDNA, and the *PfI23II* restriction site is absent in both BAC plasmid and zy30 genome. Firstly, 1–2 μg of pBeloBAC11 plasmid was digested with the restriction enzyme *BamHI* for 2 h at 30°C and purified using a Gel Extraction Kit (Omega Bio-Tek, Guangzhou, China). Then, seven overlapping PCR fragments (IBV-1 to IBV-7) were assembled consecutively into pBeloBAC11 plasmids by using the *BamHI* restriction enzyme. In fragment IBV-7, a silent substitution (A → G) was introduced at nucleotide (nt) position 25,836 using the TaKaRa MutanBEST Kit (Takara Bio-Inc., Dalian, China) according to the manufacturer’s instructions. Meanwhile, IBV-1 fragment retains a *PfI23II* restriction site close to their 5′ end and IBV-7 fragment retains a *BamHI* restriction site close to their 3′ end. Then, the HB fragment was assembled behind the 3′ terminus of IBV-7 using the *BamHI* restriction enzyme. Finally, the CMV promoter fragment was cloned in front of the 5′ terminus of IBV-1 using the *PfI23II* restriction enzyme. Thus, a cDNA clone carrying the full-length zy30 genomic cDNA sequence was constructed and designated as the BAC-zy30 plasmid. The BAC- zy30 plasmid was further characterized by restriction enzyme digestion and agarose gel electrophoresis. Meanwhile, the N fragment containing the zy30 strain N gene, a 3′ untranslated region (UTR), and a 30 nt poly(A) tail at the 3′ end was amplified using NF and NR primers and cloned into the pcDNA3.1(+) vector to generate pCDNA3.1-N plasmid construct. The homologous arms cloning method was performed by using the SE Seamless Cloning and Assembly Kit (Beijing Zoman Biotechnology Co., Ltd., Beijing, China), according to the manufacturer’s instructions.

### Generation of Three Recombinant BAC Plasmids With Mutated S2 Subunit of the IBV zy30 Strain

After comparing the complete genome sequence of the zy30 and zy100 strains, two site differences in the S2 subunits were observed including an N1038S substitution and last nine amino acid deletions at 1,154–1,162 aa (EQYRPKKSV) in the C-terminal tail of the zy100 genome sequence (Figure 1D). Therefore, one recombinant BAC plasmid carrying both of the mutations was constructed, and two additional recombinant BAC plasmids carrying either N1038S substitution or ^1154^EQTRPKKSV^1162^ deletion in the cDNA sequence of zy30 were constructed based on BAC-zy30 plasmid.

Firstly, a recombinant plasmid with a complete S gene deletion (20,364–23,909 nt) was constructed. For this purpose, the IBV-8 fragment at 17,390–20,348 nt of the zy30 genome cDNA carried an additional 34 bp homologous sequence relative to 23,909–23,942 nt of zy30 and an additional *PfI23II* restriction enzyme site at 3′ terminus (Table 1). Then, the BAC- zy30 plasmid was double-digested with the restriction enzymes of *AvrII* (17,415 nt) and *PmeI* (23,912 nt), and ligated with the IBV-8 PCR fragment. Thus, an intermediate plasmid, BAC-zy30-S^△^, with a complete S gene sequence deletion was obtained (Figure 1B). Secondly, three plasmids containing the S gene with substitutions of A to G at 23,486 nt and/or G to T at 23,833 nt were constructed. For this, the S gene at 20,364–23,909 nt of the zy30 genome cDNA sequence was amplified and cloned into a pMD 19-T Vector (Takara Bio-Inc., Dalian, China) and the mutations were introduced using TaKaRa MutanBEST Kit (Takara Bio-Inc., Dalian, China); the resulting plasmids were named as pS-G, pS-T, and pS-GT (Figure 1B). Finally, the S gene fragment carrying the A to G substitution at 23,486 nt and/or the G to T substitution at 23,833 nt were introduced into the corresponding region of the intermediate plasmid BAC-zy30-S^△^ using the *PfI23II* restriction enzyme to obtain the three final recombinant BAC plasmids, BAC-zy30S2-N1038S, BAC-zy30S2-CT9^△^, and BAC-zy30S2-N1038S-CT9^△^ (Figure 1C).

### Recovery of Infectious Virus

All virus rescue experiments were performed on BHK-21 and CEK cells. Briefly, confluent monolayers of BHK-21 cells (60 × 15 mm cell culture dishes, in triplicate) were transfected with 10 μg of either BAC-zy30, BAC-zy30S2-N1038S-CT9^△^, BAC-zy30S2-N1038S, or BAC-zy30S2-CT9^△^ plasmids together with 1 μg of pCDNA3.1-N using the Lipofectamine™ 3000 Transfection Reagent (Thermo Fisher Scientific Inc., Shanghai, China). An empty BAC plasmid was used as a control. After 36 h, the cells and supernatants were collected and inoculated into the CEK cell cultures. The cell mixtures were collected after 48 h and labeled as rzy30, rzy30S2-N1038S-CT9^△^, rzy30S2-N1038S, or rzy30S2-CT9^△^ virus, respectively. The success rescue of IBV was verified by RT-PCR amplification of the IBV N gene, as previously described (Zou et al., 2010).

The cDNA fragment of the recovered rzy3 strain containing the silent nucleotide substitution (24,991–27,660 nt) was amplified by RT-PCR with selected primer pairs (5ʹGGTGTTTGGTTTTACAAGCG3ʹ and 5ʹCTTGACGTCTCCAGTACCCAT3ʹ) at 25,500–26,148 nt and sequenced. The recovered rzy30S2-N1038S-CT9^△^, rzy30S2-N1038S, and rzy30S2-CT9^△^ strains containing the DNA fragment at 23,272–24,021 nt, which contained corresponding substitutions in the S2 subunit, were amplified by RT-PCR with primer pairs (5ʹAGTGCCTGTTAATGGTAGAGG3ʹ and 5ʹGCCAAAGGAGCA GCCTAGA3ʹ) and Sanger sequenced.

### Quantitative Real-Time RT-PCR Validation

The primer pairs (5′GGTAAGCATTGAGTGTTGTGGTGA3′ and 5′GGTTCTGGTGCCTCTGGAAACA3′) were designed and synthesized to amplify the IBV fragment at 2,616–2,777 nt for the measurement of viral RNA copies by RT-qPCR. To prepare the RT-qPCR standard plasmid, PCR fragments were amplified and inserted into the pMD19-T Vector (Takara Bio-Inc., Dalian, China). The plasmid was 10-fold serially diluted ranging from 1.0 × 10^1^ copies/μl to 1.0 × 10^6^ copies/μl. RT-qPCR was performed in a 20 μl reaction mix consisting of 0.8 μl of the forward and reverse primers (10 pmol/μl), 7.4 μl RNase free ddH2O, 1 μl DNA template, and 10 μl SYBR Premix Ex Taq TM II (Takara Bio-Inc., Dalian, China). The cycling conditions were 95°C for 30 s, then 40 cycles of 95°C for 10 s, 60°C for 15 s, and 72°C for 20 s.

### Biological Characterization of Rescued IBV Strains *in vitro*

The Tissue Culture Infectious Dose 50% (TCID_50_) titers of zy30, rzy30, rzy30S2-N1038S-CT9^△^, rzy30S2-N1038S, and rzy30S2-CT9^△^ in CEK cells were determined according to Reed and Muench method (Reed, 1938). For the determination of the replication kinetics of rescued virus in CEK cells, CEK cells were inoculated with either rzy30, rzy30S2-N1038S-CT9^△^, rzy30S2-N1038S, rzy30S2-CT9^△^, or zy30 containing 100 TCID_50_ of virus. After 45 min of virus adsorption at 37°C, cells were washed with DMEM and continuously cultured in DMEM supplemented with 1% FBS at 37°C. The supernatant was collected from the CEK cells at time intervals of 12, 24, 36, and 48 h post-inoculation (hpi) and stored until further analysis. Viral RNA was extracted, and the viral RNA copies were determined by RT-qPCR following the method established above.

### Pathogenicity Studies

A total of 116 7-day-old SPF chickens were randomly divided into six groups (I–V: *n* = 21 chickens in each group, VI: *n* = 11 chickens). Group I, II, III, IV, and V were challenged with 10^3^ TCID_50_ of rzy30S2-N1038S-CT9^△^, rzy30S2-N1038S, rzy30S2-CT9^△^, rzy30, and parental zy30 strain in a 0.2 ml preparation *via* intraocular and intranasal routes, respectively. Chickens in Group VI were inoculated with sterilized DMEM only.

**Figure 2:**
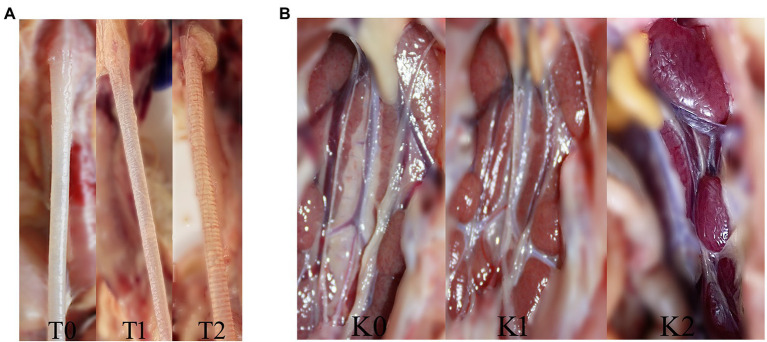
The trachea and kidney lesions in chickens challenged with IBV. **(A)** The trachea lesion and gross lesion scores: T0 (0 points) for normal, T1 (1 point) for abundant mucus and minor bleeding in the trachea, and T2 (2 points) for large hemorrhage in the trachea. **(B)** The kidney lesion and gross lesion scores: K0 (0 points) for normal, K1 (1 point) for minor kidney swelling, and K2 (2 points) for visible kidney swelling.

The clinical signs, including sneezing, tracheal rales, chills, and increased water intake in group I–V (10 chickens from each group), were observed until 14 day post-challenge (dpc). For the remaining 11 chickens in each group, eight chickens were monitored for tissue lesion development and subjected to viral RNA load quantification, and three chickens were subjected to microscopic observation. At 7 dpc and 14 dpc, four chickens in each group were randomly selected for euthanasia and necropsy. The gross lesions of the trachea and kidney in all the groups were recorded and scored by calculating the mean lesion scores (MLS). Additionally, trachea and kidney samples were carefully collected for the detection of viral loads by RT-qPCR. Gross lesions in the trachea were scored from 0 to 2 as follows: 0- normal; 1- abundant mucus and minor bleeds in the trachea; and 2- large hemorrhage in the trachea. In the kidney, the scoring system was as follows: 0- normal; 1- minor kidney swelling; and 2- visible kidney swelling (Figures 2A,B). At 7 dpc, three chickens in each group were euthanized for microscopic observation of the tissue lesions. Their trachea and kidney were collected and preserved in 10% neutral formalin embedded in paraffin for 24 h and stained with hematoxylin and eosin (HE), followed by microscopic observations.

### Statistical Analysis

Data analysis of TCID_50_ titer, lesion scores, and RT-qPCR was carried out in IBM SPSS Statistics 22 software with the Bonferroni test in ANOVA method (normal distribution/homoscedasticity) or all pairwise in Kruskal-Wallis one-way ANOVA method (non-parametric). Probability (*p*) values of <0.05 was considered statistically significant difference, *p* < 0.01 was considered highly significant difference.

### Ethics Statement

All animal experiments were conducted in compliance with protocols approved by Sichuan Provincial Laboratory Animal Management Committee [Permit Number: SYXK (Sichuan) 2019-187]. The protocols for this experiment were performed according to the guidelines of the Ethics and Animal Welfare Committee (EAWC) of the Sichuan Agricultural University. The birds were euthanized *via* cervical dislocation or *via* administration of intravenous sodium pentobarbital (100 mg/kg) by a trained technician during the experimental period as approved by the EAWC.

## Results

### Successful Construction of a Genomic cDNA of IBV zy30 Strain and Modification of S2 Subunit in the BAC

**Figure 3:**
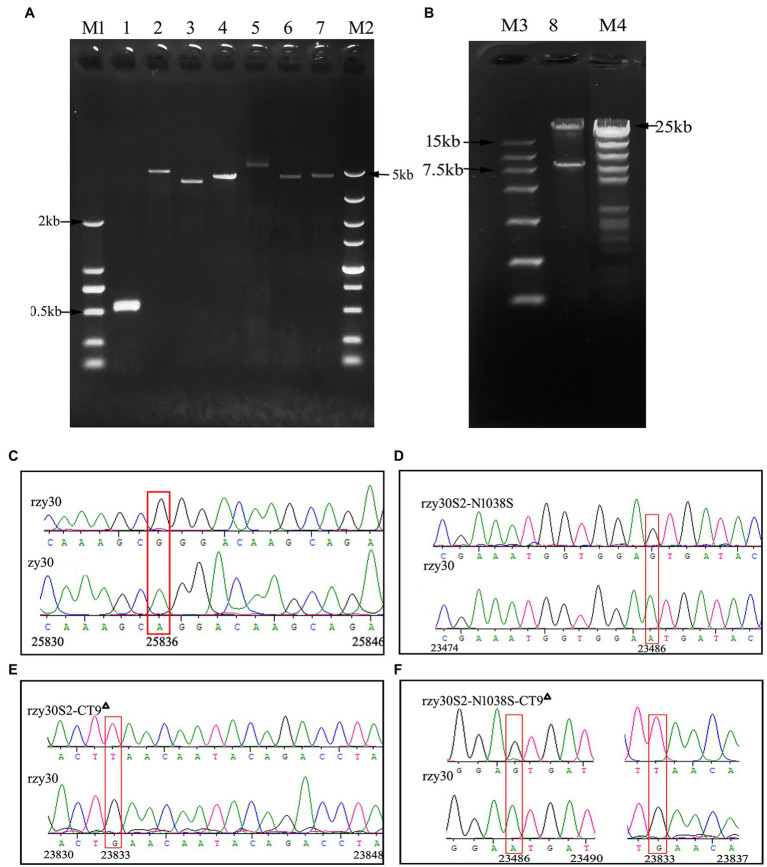
The recovered rzy30 based on BAC plasmid were confirmed. **(A)** Lane 1 to Lane 7 represent purified PCR fragments (IBV-1, IBV-2, IBV-3, IBV-4, IBV-5, IBV-6, and IBV-7) of zy30 genome used for the clone. Lane M1 and M2 represent the 2 kb and 5 kb DNA ladder, respectively. **(B)** Plasmid BAC-zy30 (Lane 8) was cleaved into two segments of approximately 28 kb and 7.6 kb in length by *BamHI* restriction enzyme digestion. Lane M3 and M4 represent the 15 kb and 23 kb DNA ladder, respectively. **(C)** The silent substitution (A to G) was introduced at 25,386 nt to distinguish rzy30 from zy30 as shown in the red box. **(D)** The mutation at 23,486 nt (A to G) was identified in rzy30S2-N1038S. **(E)** The mutation at 23,833 nt (G to T) was identified in rzy30S2-CT9^△^. **(F)** The mutations at 23,486 nt (A to G) and 23,833 nt (G to T) were identified in rzy30S2-N1038S-CT9^△^.

The full genomic cDNA of IBV zy30 strains was amplified as seven overlapping PCR fragments (IBV1-7), which were sequentially assembled into pBeloBAC plasmid utilizing two restriction enzymes and homologous arms cloning method (Figure 3A). Next, a recombinant BAC-zy30 plasmid, of approximately 35 kb in length, was constructed, containing the genomic cDNA of IBV zy30 strain between the CMV promoter and HB terminus. As shown in Figure 3B, the recombinant BAC-zy30 plasmid was identified as 7.6 kb and 28 kb fragments through *BamHI* restriction enzyme digestion. There was no mutation in the BAC-zy30 plasmid.

In order to generate mutations in S2 subunit, an intermediate plasmid BAC-zy30-S^△^ was constructed. It showed complete deletion of the S gene sequence at 20,364–23,909 nt. The exact mutation sites in the three plasmids pS-G, pS-T, and pS-GT were also confirmed by sequencing. The S gene fragment in pS-G, pS-T, and pS-GT was then inserted into the intermediate plasmid BAC-zy30-S^△^, respectively, and thus, obtain three final recombinant BAC plasmids, BAC-zy30S2-N1038S, BAC-zy30S2-CT9^△^, and BAC-zy30S2-N1038S-CT9^△^ (Figure 1C). Sequencing of S2 genes in recombinant BAC plasmids revealed the corresponding mutations (Data not shown).

### All Recombinant Viruses Were Successfully Rescued

The BHK-21 cell supernatant was harvested at 36 h post-transfection and propagated in CEK cells. Syncytia, the typical cytopathic effect (CPE) of IBV infection was observed in CEK cells at 24 h post-infection, and RT-PCR detection of N gene of IBV was also positive in the CEK culture. So, all the recombinant viruses were successfully rescued and named as rzy30, rzy30S2-N1038S, rzy30S2-CT9^△^, and rzy30S2-N1038S-CT9^△^ strain, respectively. After sequencing the mutation region, we observed a nucleotide change of A to G at 25,836 nt in the rzy30 strain (Figure 3C). For the other three recombinant viruses, the substitution of A to G at 23,486 nt in the rzy30S2-N1038S strain and the substitution of G to T at 23,833 nt in the rzy30S2-CT9^△^ strain was verified (Figures 3D,E), and the two substitutions were also confirmed in the rzy30S2-N1038S-CT9^△^ strain (Figure 3F).

### The Biological Characteristics of Rescued rzy30 Strain Are Similar to Parental zy30

A quantitative Real-Time RT-PCR (RT-qPCR) method for IBV viral nucleic acid was successfully established. The standard curves were plotted with the log of target gene copy number on the *x* axis and cycle threshold (*C*t) value on the *y* axis. The RT-qPCR reaction efficiency as calculated from the slope was 100.0%, with a correlation coefficient (*R*^2^) of 0.99 and a conclusion equation of *Y* = −3.4168LgX + 10.51. The actual standard curve for RT-qPCR was shown in Supplementary Figure 1.

**Figure 4:**
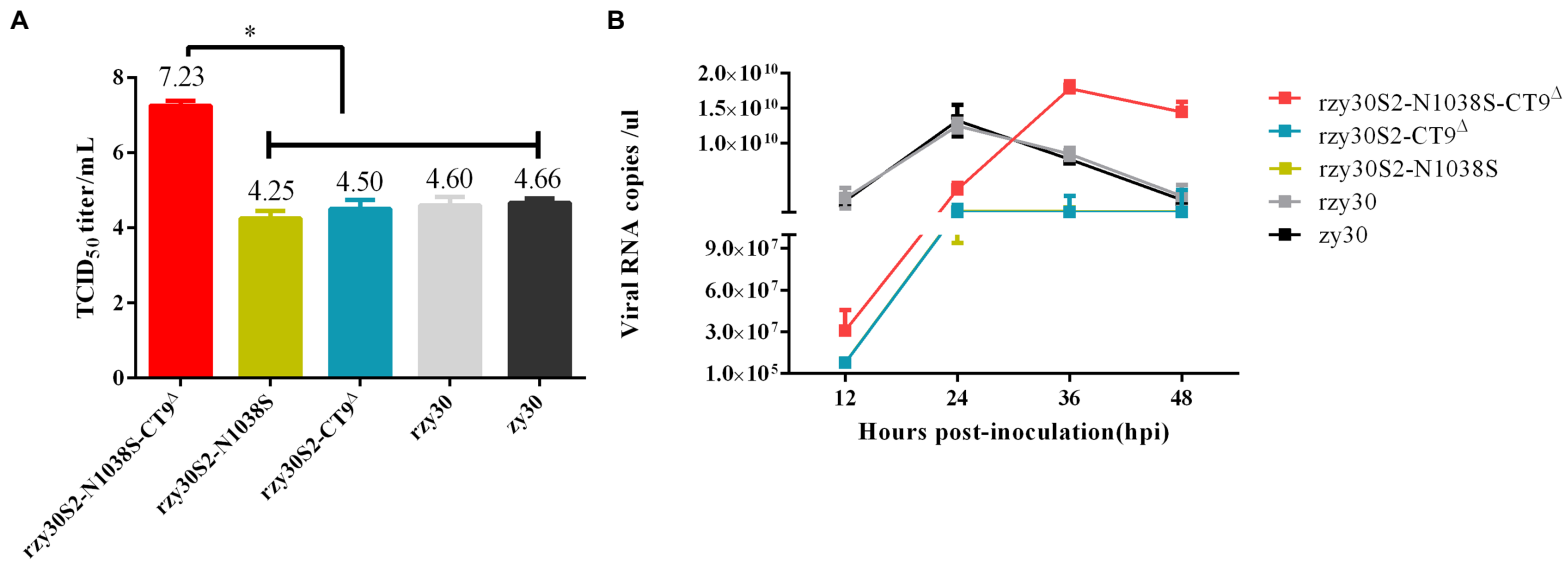
The biological characteristics of rescued strains *in vitro*. The TCID50 titer **(A)** and viral RNA copies/μl **(B)** in CEK cells after infection with rzy30S2-N1038S-CT9^△^, rzy30S2-N1038S, rzy30S2-CT9^△^, rzy30, and zy30 strains. The Bonferroni test of ANOVA method was used to analyze the statistical difference of TCID50 titer **(A)** between different groups. ^*^Significant difference between the analyzed group with other groups ( *p* < 0.05).

To observe whether the recombinant rzy30 virus generated through BAC-based reverse genetics technique exhibited the same biological characteristics *in vitro* as the parental zy30 strain, their TCID_50_ titers and growth kinetics were analyzed. The rzy30 and zy30 strains exhibited similar biological properties in CEK cells because they had similar TCID_50_ titers (Figure 4A) and growth kinetics (Figure 4B).

**Figure 5:**
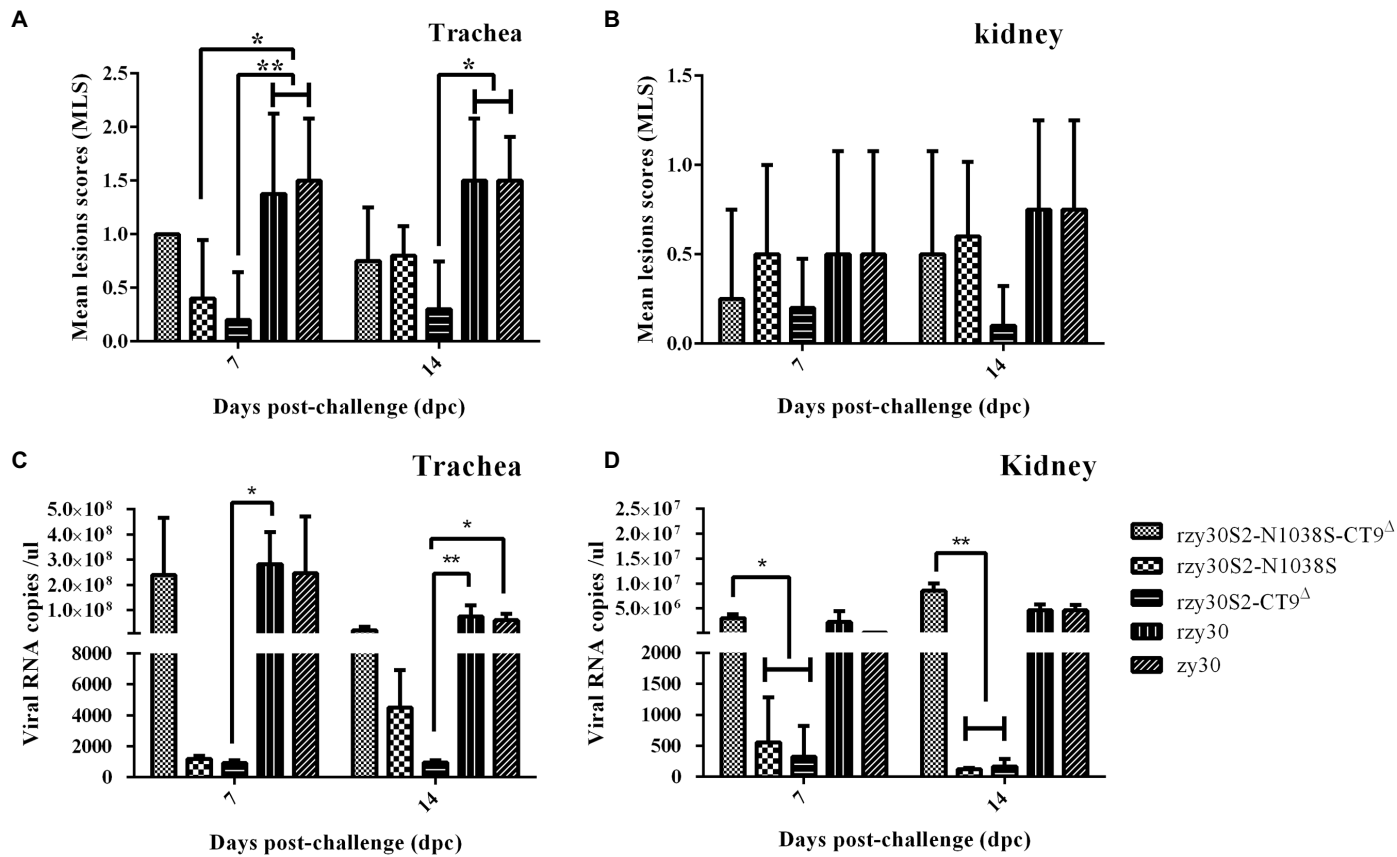
Pathogenicity results of rzy30S2-N1038S-CT9^△^, rzy30S2-N1038S, rzy30S2-CT9^△^, rzy30, and zy30 strains in SPF chickens. The MLS of trachea **(A)** and kidney **(B)** from chickens infected with IBV strains. The viral RNA copies of trachea **(C)** and kidney **(D)** from chickens infected with IBV strains. Statistical differences between different groups were determined by using the Bonferroni test in ANOVA method for the analysis of MLS of trachea **(A)** and kidney **(B)**, and the all pairwise in Kruskal-Wallis one-way ANOVA method for the analysis of the viral RNA copies of trachea **(C)** and kidney **(D)**. ^*^Significant difference between the analyzed group with other groups ( *p* < 0.05) and ^**^p < 0.01.

To observe the pathogenicity of rescued rzy30 virus in SPF chickens, one-week-old SPF chickens were infected with either the rzy30 (group IV) or the parental zy30 strain (group V). At 2–3 dpc, the infected chickens in the two groups began to display clinical symptoms, including sneezing, listlessness, huddling, and an overall morbidity of 62.5% (Table 2). The survival rate of the two groups infected with IBV was 100% throughout the 14 dpc. The rzy30 and zy30 strains caused similar trachea and kidney lesions in chickens during the 14 dpc (Figures 5A,B). In detail, the trachea MLS of chickens infected with rzy30 strain at 7 and 14 dpc were 1.375 and 1.50, respectively, which were 1.50 and 1.50 in chickens infected with zy30 strain. In addition, the kidney MLS of chickens infected with the rzy30 and zy30 strains at 7 dpc and 14 dpc were 0.50 and 0.75, respectively. The viral RNA loads in the trachea and kidneys of chickens infected with rzy30 were consistent with that of the zy30 strain at 7 dpc and 14 dpc. Pathological changes were not observed in the control group (group VI).

**Table 2:** Comparison of pathogenicity between rescued IBV and zy30 strains.

Strain	Morbidity (%)	Survival rate (%)
rzy30S2-N1038S-CT9^△^	25	100
rzy30S2-N1068S	30	100
rzy30S2-CT9^△^	20	100
rzy30	62.5	100
zy30	62.5	100
Control group	0	100

**Figure 6:**
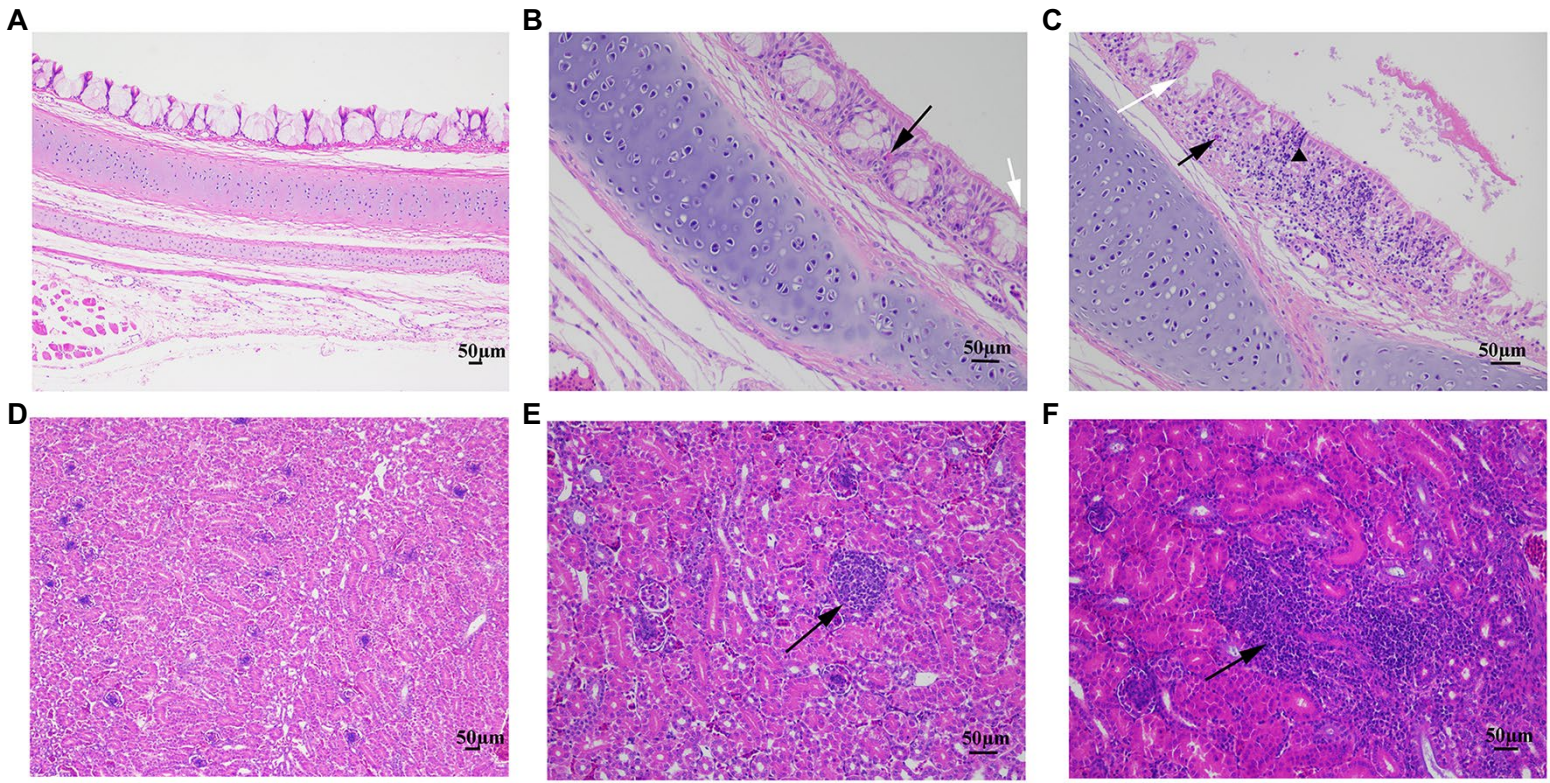
The microscopic lesions in the trachea and kidney of chickens challenged with rescued virus and zy30 strains. **(A)** Normal trachea. **(B)** Slight loss of cilia (white arrow) and bleeding (black trigon) in the ciliated epithelial cells. **(C)** Bleeding (black arrow), infiltration of lymphoid cells (black trigon), and necrosis (white arrow) in the tracheal ciliated epithelial cells following the challenge. **(D)** Normal kidney. **(E)** Localized infiltration of lymphoid cells in the kidneys. **(F)** Abundant infiltration of lymphoid cells in the kidneys (black arrow).

During microscopic observation in trachea and kidneys, no lesion was observed in the control group (Figures 6A,D). Microscopic lesions characterized by severe bleeding, infiltration of lymphoid cells and necrosis in the ciliated epithelial cells, and abundant infiltration of lymphoid cells in kidneys were observed in chickens infected rzy30 and zy30 strains (Figures 6C,F), and the extent of tissue lesions in this two groups was similar. These results demonstrated that the rescued rzy30 strain based on BAC system shares similar characteristics with its parental strain zy30.

### The N1038S Substitution and ^1154^EQTRPKKSV^1162^ Deletion in S2 Subunit Can Synergistically Increase Viral Replication in CEK Cells

The virus titer of rzy30S2-N1038S and rzy30S2-CT9^△^ strains was 10^4.25^ TCID_50_/ml and 10^4.50^ TCID_50_/ml (Figure 4A), respectively. Analysis of the viral RNA load in CEK cells showed that the replication of both rzy30S2-N1038S and rzy30S2-CT9^△^strains reached peak at 24 hpi, but its virus RNA copies were lower than rzy30 strain at 12–36 hpi (Figure 4B).

The virus titer of rzy30S2-N1038S-CT9^△^strain was 10^7.23^TCID_50_/ml, significantly higher than that of rzy30 strain (10^4.60^ TCID_50_/ml; *p* = 0.011; Figure 4A). Determination of the viral RNA copies showed that the replication of the rzy30 strain in CEK cells reached peak at 24 hpi, while the replication of rzy30S2-N1038S-CT9^△^ reached peak at 36 hpi. Although the replication capacity of the rzy30S2-N1038S-CT9^△^ strain lagged behind the rzy30 strain at 12–24 hpi, it showed significantly stronger replication capacity than the rzy30 strain at 36–48 hpi (Figure 4B). These results demonstrated that the N1038S substitution and ^1154^EQTRPKKSV^1162^ deletion in S2 subunit can synergistically increase viral infectivity in CEK cells.

### N1038S Substitution and ^1154^EQTRPKKSV^1162^ Deletion in S2 Subunit, Individually or Synergistically, Reduce Viral Virulence

The pathogenicity of three recombination strains was assessed. Compared with rzy30 strain group with 62.5% morbidity, the morbidity in group rzy30S2-N1038S-CT9^△^ (group I), rzy30S2-N1038S (group II), and rzy30S2-CT9^△^ (group III) was 25, 30, and 20%, respectively (Table 2). Three recombination strains decrease clinical symptoms in infected chickens during 14 days of monitoring. The rzy30S2-CT9^△^ strain could lead to lowest morbidity than that of the other groups.

Through examination of gross lesions in trachea and kidneys, three recombination strains could cause milder tissue lesion, characterized by minor mucus and bleeds in the trachea and minor kidney swelling, compared with rzy30 strain. The tissue lesion difference in each group was reflected by MLS (Figures 5A,B). The trachea MLS in chickens infected with rzy30S2-N1038S-CT9^△^, rzy30S2-N1038S, rzy30S2-CT9^△^, and rzy30 strains were 1.00, 0.40, 0.20, and 1.375 at 7 dpc, and 0.75, 0.80, 0.30, and 1.5 at 14 dpc, respectively; the kidney MLS were 0.25, 0.50, 0.20, and 0.5 at 7 dpc and 0.50, 0.60, 0.10, and 0.75 at 14 dpc, respectively. The trachea MLS of rzy30S2-N1038S strain were significantly lower than that of rzy30 strain at 7 dpc (*p* = 0.037). The trachea MLS of rzy30S2-CT9^△^ strain were significantly lower than that of rzy30 strain at 7 dpc (*p* = 0.009) and at 14 dpc (*p* = 0.027).

The viral RNA load in trachea and kidneys of chickens infected with three recombinant strains and rzy30 strain were determined (Figures 5C,D). This analysis showed the viral loads of trachea and kidneys in rzy30S2-N1038S and rzy30S2-CT9^△^ strain groups were both lower than that of rzy30 strain and rzy30S2-N1038S-CT9^△^ strain at 7 dpc and 14 dpc. The viral RNA load of trachea in rzy30S2-CT9^△^ strain group was significantly lower than that of rzy30 strain at 7 dpc (*p* = 0.021) and at 14 dpc (*p* = 0.005). The viral RNA load of kidney in the rzy30S2-N1038S strain group was significantly lower than that of rzy30S2-N1038S-CT9^△^ strain at 7 dpc (*p* = 0.018) and at 14 dpc (*p* = 0.007), and the rzy30S2-CT9^△^ strain group was significantly lower than that of rzy30S2-N1038S-CT9^△^ strain at 7 dpc (*p* = 0.027) and at 14 dpc (*p* = 0.009). The rzy30S2-N1038S-CT9^△^ strain has a slightly higher viral load of kidneys than that of rzy30 strain at 7 dpc and 14 dpc, although there was no significant difference.

The microscopic lesions in the trachea and kidneys collected from the challenged chickens were further characterized. Compared with chickens infected by rzy30 strain, chickens infected by other three recombinant strains displayed alleviated histopathological changes in fewer samples, including slight loss of cilia and bleeding in the ciliated epithelial cells and localized infiltration of lymphoid cells in kidneys (Figures 6B,E). The differences of microscopic lesions among the three recombinant strains were consistent with those of gross lesions (data not shown).

## Discussion

Infectious bronchitis virus infection often makes chickens more susceptible to secondary infections, including bacteria or other pathogens, thus increasing their mortality. Moreover, the increased genetic and antigenic distance between vaccine strains and IBV field isolates often enhance the risk of immune failure in vaccinated birds (Li et al., 2020b).

The reverse genetics system for epidemic QX-like IBV will provide a reliable platform for IBV molecular research and the development of vaccines capable of providing enhanced protection and drugs capable of controlling epidemic strains. Several approaches have been successfully applied to recover recombinant IBV *in vitro*, which include assembly of full-length genomes by cDNA ligation, using the vaccinia virus as a vector, and targeted RNA recombination for IBV. However, these methods are complex and time consuming (Zhou et al., 2013; Van Beurden et al., 2017; Zhao et al., 2019a). Recently, IBV H120 vaccine strain was successfully rescued by using the BAC reverse genetics system. However, the IBV genomic cDNA assembly method was performed by cloning several PCR fragments of large size even longer than 10 kb into a BAC plasmid, which may lead to the infidelity of nucleotide sequences (Lv et al., 2020). Therefore, methods for constructing IBV cDNA *in vitro* still lack good reproducibility and convenient operation. In this study, the plasmids BAC-zy30 and pcDNA 3.1-N (which contained N gene of IBV zy30) were co-transfected into BHK-21 cells and rescued virus was then amplified in CEK cells. It has been reported that exogenous N protein (or N mRNA) can improve the rescue efficiency of coronaviruses (Casais et al., 2001); therefore, eukaryotic expression vectors expressing N gene of IBV were co-transfected during virus rescue. The rescue process takes the advantage on avoiding RNA electroporation and blind passage in chicken embryos, yet allows for the direct observation of typical CPE which indicates the successful recovery of IBV. Notably, the biological characteristics *in vitro* and pathogenicity of the rescued rzy30 were similar to the parental strain zy30, indicating that the rescued rzy30 strain shared similar properties with its parent strain. Hence, the method for IBV rescue established in this study is reliable.

The CEK cells are the primary target cells of IBV infection *in vitro*, but the mechanisms of increasing proliferation efficiency and attenuation of IBV after continuous passage on CEK cells remain unclear. The S2 protein performs the function of membrane fusion and affects cellular tropism (Cheng et al., 2019; Jiang et al., 2020). Therefore, it was speculated that S2 protein may play an important role in IBV replication and attenuation in CEK cells.

By reverse genetics system established for zy30 strain, three mutant strains were constructed and their phenotypic changes were explored. The result showed that N1038S substitution and ^1154^EQTRPKKSV^1162^ deletion hindered the virus replication at 12–36 hpi but did not significantly decrease TCID^50^ titer (Figures 4, 5C,D). It is possible that these two strains have overcome the suppression of natural immunity in the late stage of infection. While the N1038S substitution and ^1154^EQTRPKKSV^1162^ deletion in the S2 protein had a synergistic effect in enhancing the replicative capacity of the recombinant strain in CEK cells, with a higher TCID_50_ titer and viral RNA load in CEK cell, compared with either of the single mutation and rzy30 strain (Figures 4, 5C,D). Our unpublished data showed that the S2 subunit determines CEK cell tropism (data not shown). Therefore, the S2 subunit is not only crucial for the very first step in virus infection, but also contributes to virus-cell fusion (Yamada et al., 2009). It has been reported recently that the L581F substitution in the S2 subunit promoted adaptation in CEK cells (Jiang et al., 2020), but no difference was observed between zy30 and zy100, possibly because this site-related adaption to CEK cells varies between different strains.

Current studies indicated that IBV virulence was influenced by multiple genes, including S gene, 3 gene, 5a gene, and 1a gene (Zhao et al., 2019a,b, 2020). However, the key sites affecting virulence have not been reported. This study showed that the three recombinant strains exhibited decreased pathogenicity in chickens, since lower morbidity, as well as a reduction in trachea and kidneys lesions, was observed (Figures 5, 6), and the pathogenicity of the rzy30S2-CT9^△^ strain was the lowest. Therefore, each mutation lead to attenuation, and the deletion of EQTRPKKSV in the cytoplasmic tail of the S2 protein played a greater role in the attenuation process. For IBV, its replicative efficacy in CEK cells does not appear to be necessarily related to virulence, but it still affects the replication ability in chicken kidneys *in vivo*. Neither mutation could generate recombinant virus similar in virulence to SczyC100 (Xia et al., 2018); thus, other genes must also be involved in Sczy3 strain attenuation by serial passage in CEK cells.

**Figure 7:**
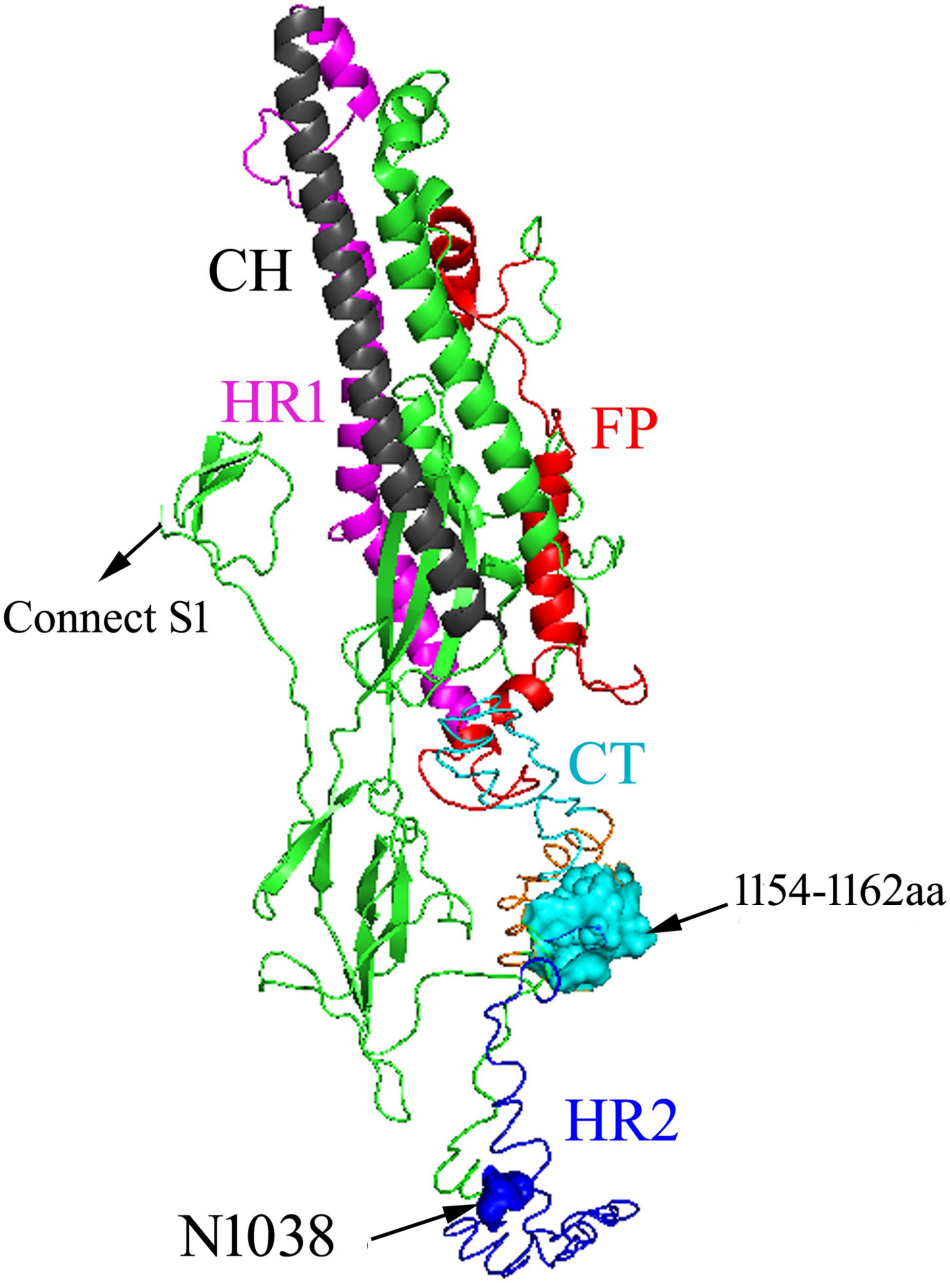
Schematic illustration of zy30 S2 protein drawn by comparison with the corresponding sequence of the M41 strain. FP: fusion peptide. HR1: heptad repeats 1. CH: central helix. HR2: heptad repeats 2. The range of FP was not determined. The HR2 was disordered. Amino acid residues at 1038 aa (blue) and 1,154–1,162 aa (cyan).

We predicted the pre-fusion structure of the S2 subunit of the IBV zy30 strain, based on the cryo-electron microscopic structure of the IBV M41 S protein (Shang et al., 2018), by homology modeling using the SWISS-MODEL server^2^ (Figure 7). The N1038S substitution was located in the HR2 region, and the 1,154–1,162 aa (EQYRPKKSV) was located in cytoplasmic tail. The disordered HR2 will be refolded into a mixture of α-helices and coils and packed into a six-helix bundle structure (6HB) with HR1 in the post-fusion structure, which is essential for the viral fusion (Shang et al., 2018; Xia et al., 2020). Similarly, a substitution of P1263L in the HR2 region of the SARS-CoV-2 S2 protein has been shown to decrease infectivity (Li et al., 2020a). In addition, the N1038 aa is predicted to be an N-glycosylation site in the IBV zy3 strain.^3^ Glycosylation of the spike protein in coronaviruses has been demonstrated to be a determinant for protein folding and receptor interactions and can mask neutralizing epitopes present on the spike protein, thereby influencing host immunity (Han et al., 2007; Tortorici et al., 2019). The IBV S protein contains multiple N-glycosylation sites, and the deletion of glycosylation sites on the S1 subunit could negate binding to host receptors and decrease virus replication in cells (Zheng et al., 2018; Parsons et al., 2019). Thus, the N1038S substitution at HR2 in the S2 subunit may affect 6-HB formation or immunogenicity *via* N-glycosylation deletion, thus decrease early replication in infected cells and reduce pathogenicity in chickens.

The C-terminal domain of the S2 protein could serve as a bridge for interaction with other viral proteins such as membrane (M) proteins and contains a variety of signal sequences (Sadasivan et al., 2017; Kumar et al., 2021). For example, the KKSV motif at the C-terminus of the S2 subunit of IBV acts as the endoplasmic reticulum (ER) retrieval signal, and an absence of the ER retrieval signal caused a growth defect at later stages of infection (Youn et al., 2005). Deletion of multiple amino acids in the cytoplasmic tail of the S protein has been shown to lead to changes in host cell coronavirus infectivity (Ujike et al., 2016; Yu et al., 2021), which in IBV zy30 strain is represented by a growth defect at early stages of infection in CEK cells and decreased virulence. However, the simultaneous N1038S substitution and the ^1154^EQTRPKKSV^1162^ deletion significantly increased virus infectivity of the CEK cell, especially in the later stages of infection. Whether this was accomplished with inhibition of viral entry or natural immune is unknown and requires further study.

## Conclusion

In conclusion, a reverse genetics system of QX genotype zy30 strain was constructed based on a bacterial artificial chromosome (BAC) system. The influence of the N1038S substitution and ^1154^EQTRPKKSV^1162^ deletion in S2 subunit on the replication and pathogenicity was evaluated. The simultaneous mutation of two sites can improve the viral proliferation efficiency in CEK cells and reduce pathogenicity, compared to rzy30 strain. The role of ^1154^EQTRPKKSV^1162^ deletion was greater in reducing pathogenicity than N1038S substitution. This research provides reference data for research on the replication and pathogenic mechanism of IBV.

## Data Availability Statement

The original contributions presented in the study are included in the article/**Supplementary Material**, further inquiries can be directed to the corresponding author.

## Ethics Statement

The animal study was reviewed and approved by Sichuan provincial Laboratory Animal Management Committee [Permit Number: SYXK (Sichuan) 2019-187].

## Author Contributions

SL contributed to the conception of the study. SL, SF, and NL contributed to the date acquisition and manuscript preparation. YS and XX helped perform the methodology and data analyses. WC helped perform the analysis with constructive discussions. MC and XH contributed to providing resources. JX and YH contributed to the supervision and writing—review and editing. All authors contributed to the article and approved the submitted version.

## Funding

This work was financially supported by the Program for Chang-jiang Scholars and Innovative Research Team in University “PCSIRT” (grant no. IRT0848).

## Conflict of Interest

The authors declare that the research was conducted in the absence of any commercial or financial relationships that could be construed as a potential conflict of interest.

## Publisher’s Note

All claims expressed in this article are solely those of the authors and do not necessarily represent those of their affiliated organizations, or those of the publisher, the editors and the reviewers. Any product that may be evaluated in this article, or claim that may be made by its manufacturer, is not guaranteed or endorsed by the publisher.

## Footnotes

^1^ https://talk.ictvonline.org

^2^ https://zhanggroup.org/I-TASSER/

^3^ www.cbs.dtu.dk/services/NetNGlyc/
