# Supplementary material for: The N1038S Substitution and 1153EQTRPKKSV1162 Deletion of the S2 Subunit of QX-Type Avian Infectious Bronchitis Virus Can Synergistically Enhance Viral Proliferation
Source: Front Microbiol. 2022 Mar 30;13:829218. doi: 10.3389/fmicb.2022.829218 (PMC9006875; doi:10.3389/fmicb.2022.829218)
Supplement: Supplementary file 1 [file Data_Sheet_1.DOCX]

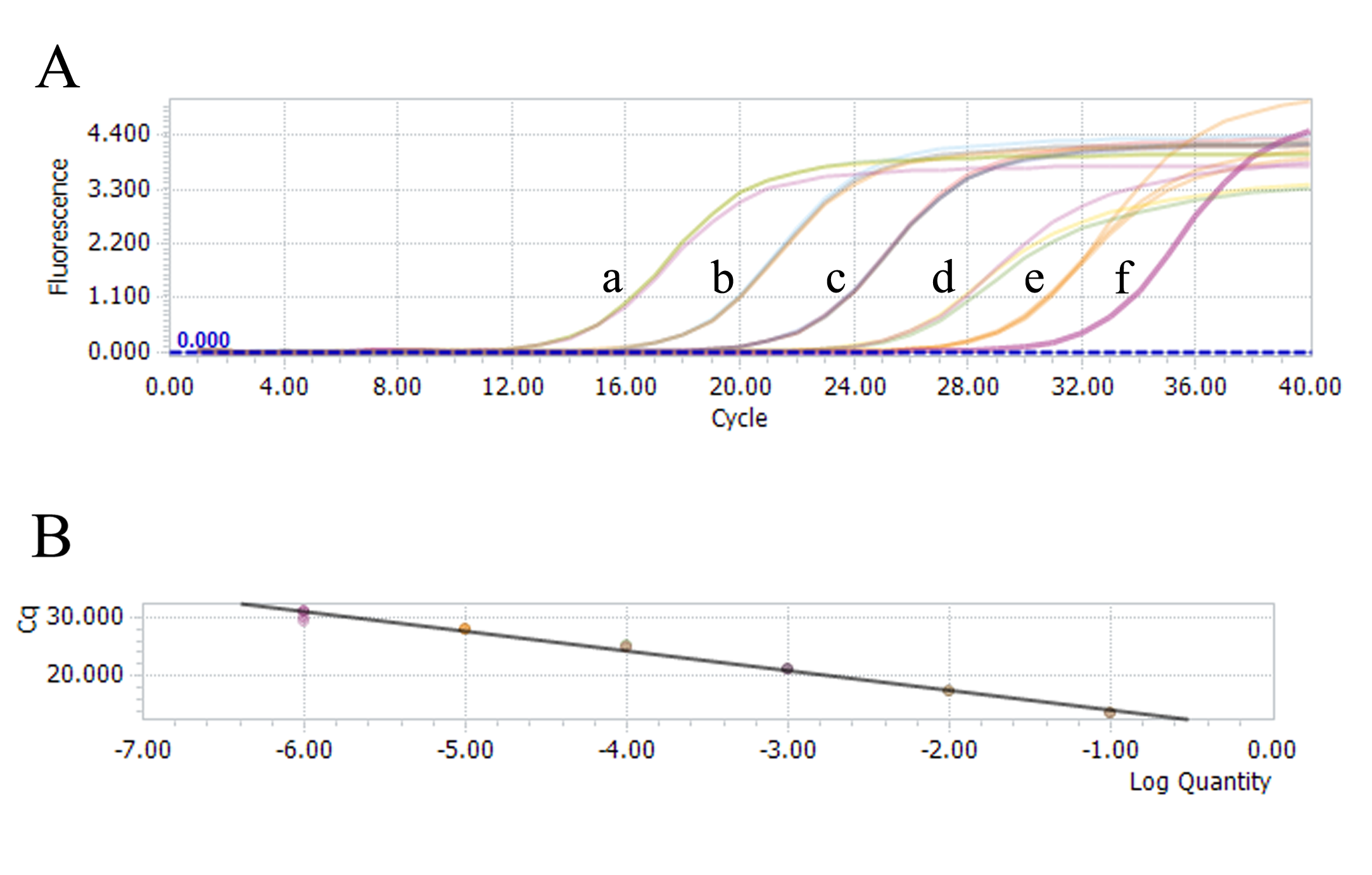


Supplementary Figure The standard curves of RT-qPCR. A: Reproducibility test of the SYBR Green I RT-qPCR assay. Each plot corresponds to a particular input target copy number ranging from 1*10^1^-1*10^6^ (a-f). B: The standard curve was generated by plotting the Cq values vs log 10 of 10-fold serial dilutions (1*10^1^-1*10^6^) of standard plasmid. An overall reaction efficiency of 100.0%, was estimated using the standard curve slope as indicated by the formula.
